# Predicting behavioral escalation in students with autism using physiological and movement data

**DOI:** 10.3389/fpsyt.2026.1850879

**Published:** 2026-10-26

**Authors:** Richard Lamb, Tosha Owens, Ikseon Choi, Shruti Kundu, Sagar Nookkarapu, Priya Ray, Amanda Kavner

**Affiliations:** 1 Department of Clinical and Administrative Pharmacy, Neurocognition Science Laboratory, College of Veterinary Medicine, College of Pharmacy, Department of Physiology and Pharmacology, University of Georgia, Athens, GA, United States; 2 Department of Special Education, Foundations, and Research, College of Education, East Carolina University, Greenville, NC, United States; 3 Nell Hodgson Woodruff School of Nursing, Emory University, Atlanta, GA, United States; 4 Neurocognition Science Laboratory, College of Veterinary Medicine, University of Georgia, Athens, GA, United States

**Keywords:** artificial intelligence, autism spectrum disorder (ASD), behavioral escalation, physiological monitoring, wearable sensors

## Abstract

**Introduction:**

Behavioral escalation in students with Autism Spectrum Disorder (ASD) presents significant challenges in educational and community settings, often leading to reactive interventions after behaviors become observable. This study investigated whether physiological and movement data obtained from wearable biometric sensors could retrospectively identify precursors of escalation and inform proactive support strategies.

**Methods:**

Physiological and movement data were collected from 30 students with ASD in naturalistic classroom environments using wearable sensors. Measures included heart rate variability (HRV), galvanic skin response (GSR), skin temperature, and accelerometer data. Behavioral observations were used to identify escalation events, and machine learning models, including Random Forest, Support Vector Machine (SVM), and Long Short-Term Memory (LSTM) networks, were trained on previously collected physiological and behavioral data to classify pre-escalation states. Model performance was evaluated retrospectively rather than through real-time deployment.

**Results:**

Among the models tested, the LSTM network achieved the highest classification accuracy (85.2%) in identifying pre-escalation states. Physiological changes associated with escalation were detectable approximately 2 to 5 minutes before observable behavioral manifestations. HRV and GSR emerged as the strongest predictive features, while inclusion of accelerometer data further improved classification performance. These findings suggest that measurable physiological patterns precede overt behavioral escalation in students with ASD.

**Discussion:**

The results support the feasibility of using wearable biometric data and machine learning approaches to retrospectively identify antecedent physiological markers of behavioral escalation. Interpreted through frameworks drawn from Applied Behavior Analysis (ABA) and Cognitive Behavioral Therapy (CBT), these physiological signals may function as both antecedents to challenging behavior and indicators of internal emotional or regulatory states. Although real-time implementation was not evaluated, the findings provide a foundation for future predictive monitoring systems designed to facilitate earlier intervention, reduce reliance on reactive or punitive responses, and promote more inclusive and emotionally responsive educational environments for neurodiverse learners.

## Introduction

Autism Spectrum Disorder (ASD) is a complex neurodevelopmental condition often characterized by differences in social communications, restricted interests, and stereotypic and repetitive behaviors such repeated vocalizations or hand-flapping (1, 2). Emotional regulation difficulties and behavioral escalations are particularly concerning in educational and community settings. Behavioral escalation involves a progressive intensification of emotional and behavioral responses, often leading to disruptive or aggressive outcomes (3). Ultimately, escalations can endanger the student and others. In severe cases, escalation may place the individual, peers, educators, caregivers, or family members at risk of physical injury and may necessitate restrictive interventions or emergency responses. These behaviors disrupt learning, strain relationships, and increase stress for both students and staff (4). Despite their impact, predicting and mitigating escalations remains difficult, especially when triggers are not immediately observable. Current strategies are largely reactive, relying on behaviors that appear too late for effective intervention (5). Recent advances in wearable technology offer a proactive alternative. Physiological signals—such as heart rate variability (HRV), galvanic skin response (GSR), and skin temperature (ST)—along with accelerometer data, can provide real-time insights into a student’s internal state (6). These biomarker signals are linked to autonomic nervous system (ANS) activity, which is critical to emotional regulation and stress responses such as those seen in behavioral escalations (7). Continuous monitoring may allow early detection of distress before behaviors escalate, enabling timely, personalized interventions that support emotional regulation in students with autism.

The use of physiological data from the autonomic nervous system response depends on a number of interacting systems to include the hypothalamus, amygdala, and prefrontal cortex (8, 9). These systems respond to stimuli and cause changes in HRV and other physiological markers which can reflect shifts in an individual’s emotional state, such as moving from a calm, socially engaged state, to a defensive or dysregulated state (10–12). In the context of autism, where difficulties with emotional regulation are common, the authors believe these physiological shifts serve as early indicators of behavioral escalation (13). Additionally, principles from Applied Behavior Analysis (ABA) provide a behavioral lens through which to interpret these physiological changes, linking them to antecedents and consequences of behavior (14). While the potential of physiological monitoring is well-recognized, its application in educational settings, particularly for students with autism, remains largely underexplored. Existing research focuses on retrospective analyses of recorded footage or applications in controlled laboratory settings, limiting the generalizability of findings to real-world environments (15–17). Moreover, few studies have integrated machine learning and multiple physiological and movement signals to develop predictive models of behavior. This gap in the literature highlights the need for research that not only examines the predictive value of these signals but also evaluates their feasibility and effectiveness in naturalistic school and community settings.

A recent large-scale investigation demonstrated the feasibility of using wearable physiological monitoring and machine learning approaches to identify agitation and behavioral dysregulation in individuals with autism and intellectual and developmental disabilities (18). While this prior work established the potential utility of wearable biomarkers for detecting behavioral risk, the present study extends the literature in several important ways. First, the current investigation focuses specifically on naturalistic school environments rather than clinical or residential settings. Second, the study integrates multiple autonomic and movement signals collected continuously during routine educational activities. Third, the work evaluates predictive windows preceding observed behavioral escalation events, with the goal of informing proactive educational intervention. Taken together, these distinctions position the current study as an extension of existing behavioral prediction literature into everyday educational practice.

This study addresses a critical gap by investigating the use of heterogeneous physiological signals—HRV, GSR, skin temperature, and accelerometer data—to predict behavioral escalation in students with autism. Continuous data were collected via wearable sensors during regular school activities to identify patterns preceding escalation. These biomarkers were used to train machine learning models intended for future real-time prediction (19–23). The goal is to develop a system that alerts educators to early signs of distress, enabling proactive interventions before behaviors become disruptive. Additional behavior management relies on subjective observations, which are often delayed and inconsistent. In contrast, physiological monitoring offers objective, immediate insights, aligning with personalized education practices. For students who struggle to express internal states, this approach makes the invisible visible, supporting more responsive and inclusive environments. Beyond the classroom, early detection can reduce restrictive interventions and ease the burden on caregivers. This research contributes to a broader shift toward compassionate, data-driven practices in special education (24). It also responds to the limitations of Applied Behavior Analysis (ABA), which may overlook internalized or subtle signs of distress underscoring the need for real-time, objective monitoring systems (25).

### Autonomic nervous system monitoring

Physiological monitoring of the ANS offers a promising avenue for addressing this gap (26). Research has demonstrated that changes in HRV, GSR, and ST often precede observable behavioral manifestations of stress or agitation (20, 27–29). Additionally, physiological indicators of internal restlessness and hyperactivity have been shown to emerge prior to observable behavioral manifestations, further supporting the use of biometric monitoring for early identification of escalating behavioral states (30). By continuously monitoring these biomarkers, it is possible to detect early physiological changes that may signal an impending escalation, thereby enabling preemptive intervention (31). However, the feasibility of implementing such monitoring systems in educational settings has been questioned. However, recent advancements in computational capabilities and increased wearable senor affordability have significantly reduced the barriers to entry and use. Modern wearable devices are capable of collecting high-resolution physiological and movement data in a non-invasive and unobtrusive manner for extended periods (often hours or days), making them suitable for continuous use in school, clinical, and community environments (22, 32). These devices have also coincided with broader trends in educational technology and personalized learning, where data-driven approaches are increasingly employed to support unique needs (33). For students with ASD, who may experience difficulties in articulating their internal states, wearable sensors offer a valuable means of accessing otherwise inaccessible information about their emotional and physiological status (34). Machine learning algorithms, such as Random Forests, Support Vector Machines (SVM), and Long Short-Term Memory (LSTM) networks, are well-suited to analyzing complex, high-dimensional datasets associated with the physiological responses associated with behavioral escalation (35, 36). The integration of physiological monitoring and machine learning into behavioral support systems also holds substantial practical implications for educators and caregivers by facilitating earlier, more targeted interventions that may prevent escalation and reduce behavioral disruption (37). A predictive system capable of alerting educators and community care personnel to early signs of escalation would enable more timely and targeted interventions, thereby reducing the frequency and severity of behavioral incidents (38).

In addition to its practical and theoretical contributions, this study addresses a critical gap in the existing literature. While prior research has explored the use of physiological data in clinical and laboratory settings, there are few studies examining the application of these methods in naturalistic educational environments (39–41). Moreover, few studies have focused specifically on the use of multimodal heterogenous physiological signal and movement data to predict behavioral escalation in students with ASD (42). Students with ASD are disproportionately affected by exclusionary disciplinary practices, often as a result of misunderstood or mismanaged behaviors by educators and community members (43). By providing educators and community members with objective, real-time data on students’ physiological states, this study aims to reduce reliance on punitive or reactive measures and promote more proactive approaches to behavior management. The development of predictive systems using ANS and functional central nervous system measures such as function near infrared spectroscopy support early intervention. By investigating the predictive value of physiological data such HRV, GSR, ST, and accelerometer data for behavioral escalation in students with ASD, this study aims to contribute to the development of innovative, data-informed practices that enhance educational outcomes and promote the well-being of neurodiverse learners.

### Purpose, research questions and hypotheses

The primary aim of this study is to evaluate the feasibility and effectiveness of using heterogeneous autonomic nervous system (ANS) signals—HRV, GSR, skin temperature (ST), and accelerometer data—to predict behavioral escalation in students with ASD. By collecting real-time physiological and movement data during regular school activities, the study seeks to identify patterns that precede escalation and train machine learning models for early detection (19, 44). The goal is to develop a system that alerts educators to early signs of distress, enabling proactive interventions before behaviors become disruptive. Behavioral escalation often results from internal stressors and environmental triggers that are not easily observed. Traditional behavioral observation methods are reactive and may miss early physiological indicators of dysregulation (45). Wearable sensors and machine learning offer a way to bridge this gap, supporting timely, individualized responses. A secondary goal is to determine which combination of physiological and movement features yields the highest predictive accuracy. While individual signals have been studied, their combined predictive value remains underexplored (46, 47). The study also examines how far in advance escalation can be predicted, informing intervention timing. These findings will guide future applications of physiological monitoring and AI in special education and behavioral support. To address these purposes the following research questions have been developed.

Research Question 1, Which physiological signals—heart rate variability (HRV), galvanic skin response (GSR), skin temperature (ST), and movement data predict behavioral escalation in students with autism? This question explores the potential of wearable sensor data to shift behavioral management from reactive to proactive. HRV, GSR, and ST are direct indicators of autonomic nervous system activity, which is closely tied to emotional regulation (48). Combined with accelerometer data, these signals may offer early warning signs of distress before behaviors become observable. Understanding their predictive capacity is essential for developing real-time monitoring systems that support timely, individualized interventions in educational settings. Hypothesis 1: Physiological signals (HRV, GSR, and ST), when combined with accelerometer data, can significantly predict behavioral escalation events in students with autism.

Research Question 2, which combination of physiological signal features provides the highest predictive accuracy for behavioral escalation? This question explores how combining multiple physiological and movement signals—HRV, GSR, ST, and accelerometer data—can enhance the performance of predictive models. While individual signals have shown promise in detecting stress and arousal, their combined use may yield greater accuracy. Identifying the most informative feature sets is essential for building efficient, real-world monitoring systems. This also informs the design of wearable devices by potentially reducing the number of required sensors, improving comfort and usability. The question aligns with machine learning best practices, where feature selection is key to model optimization. Hypothesis 2: Multimodal signals (HRV, GSR, ST, and movement data) will yield higher predictive accuracy than any single signal alone.

Research Question 3, how early can behavioral escalation be predicted before observable behaviors occur? This question addresses the timing of prediction, which is critical for practical intervention. A predictive system must not only be accurate but also provide sufficient lead time for educators to act. If escalation can be reliably predicted 2–10 minutes in advance, staff can implement de-escalation strategies to prevent disruptive outcomes. This also deepens our understanding of the physiological precursors to dysregulation in students with autism. Hypothesis 3: Behavioral escalation can be predicted using physiological and movement data at least 2–10 minutes before observable signs appear.

### Theoretical framework

This study is grounded in an integrated theoretical framework combining Cognitive Behavioral Therapy (CBT) and Applied Behavior Analysis (ABA) to understand and predict behavioral escalation in students with ASD. While traditionally applied in separate contexts, their integration offers a comprehensive lens to examine both internal emotional processes and observable behaviors. ABA, rooted in operant conditioning, focuses on the functional relationship between antecedents, behaviors, and consequences (15, 49). It is widely used to identify triggers and reinforce adaptive behaviors in individuals with autism (50). The escalation cycle, conceptualized as antecedent-behavior-consequence (ABC), is central to understanding behavioral outbursts. CBT complements this by addressing internal cognitive and emotional processes that may lead to dysregulation (51, 52). Though traditionally used with verbal populations, its principles—such as identifying emotional triggers and developing self-regulation—can be adapted for nonverbal individuals with ASD (53). In this study, physiological signals like HRV, GSR, and ST are treated as somatic markers of emotional arousal (92).

**Figure 1** illustrates the integration of Cognitive Behavioral Therapy (CBT) and Applied Behavior Analysis (ABA) within a physiological monitoring framework for predicting behavioral escalation in students with ASD. This dual framework supports a predictive model that links real-time physiological data to both internal states (CBT) and behavioral outcomes (ABA), enhancing ecological validity and enabling data-driven, individualized interventions in educational settings. The model underscores the value of integrating internal state monitoring with behavioral observation, offering a holistic, data-driven approach to emotional and behavioral support in educational settings.

**Figure 1:**
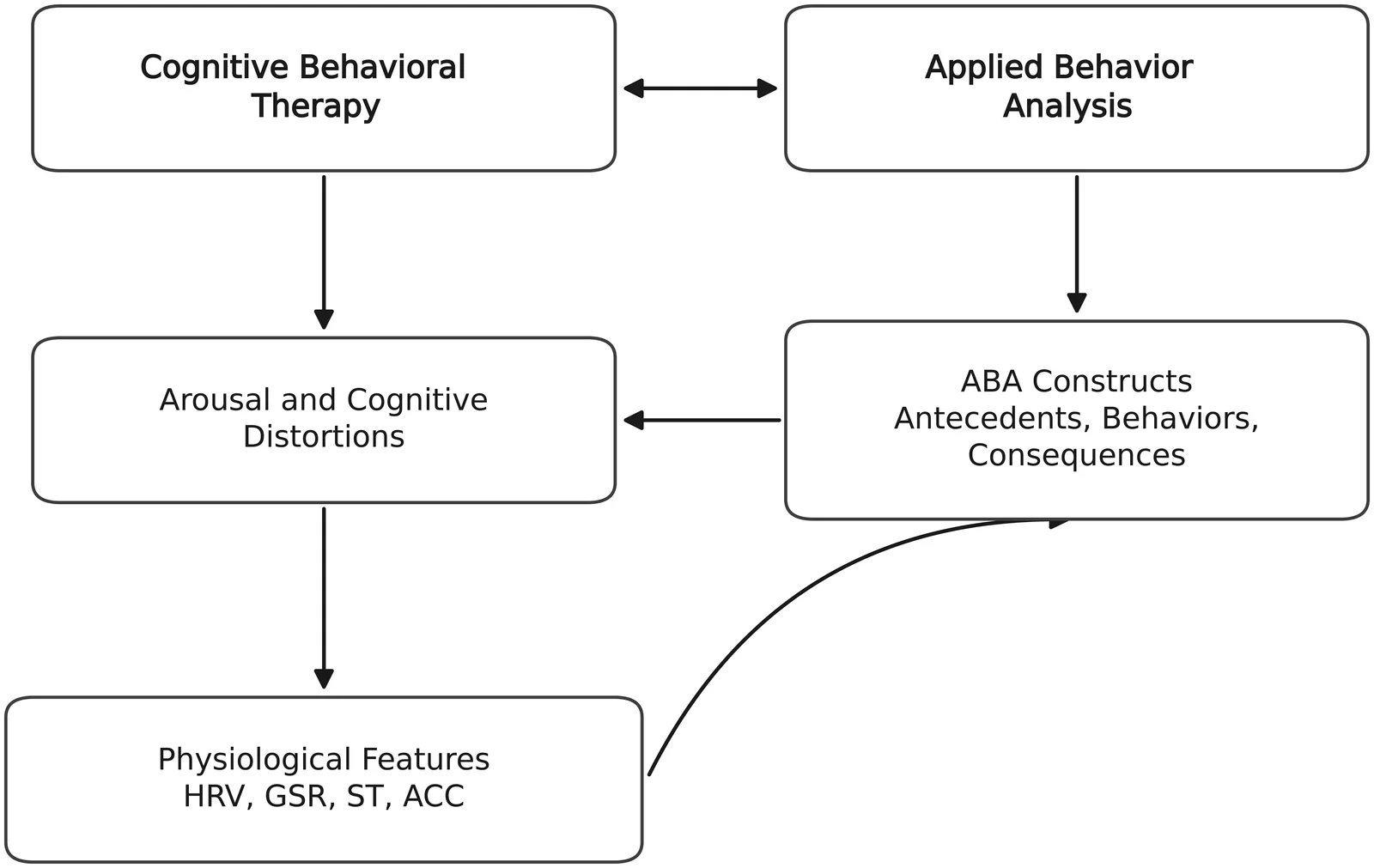
CBT-ABA framework.

### Review of the literature

Behavioral escalation in ASD has traditionally been addressed through functional behavior assessment and ABA-based interventions. While effective, these approaches are primarily reactive and do not directly assess internal physiological states preceding escalation (24, 54–56). Please see **Figure 2** for an example escalation cycle.

**Figure 2:**
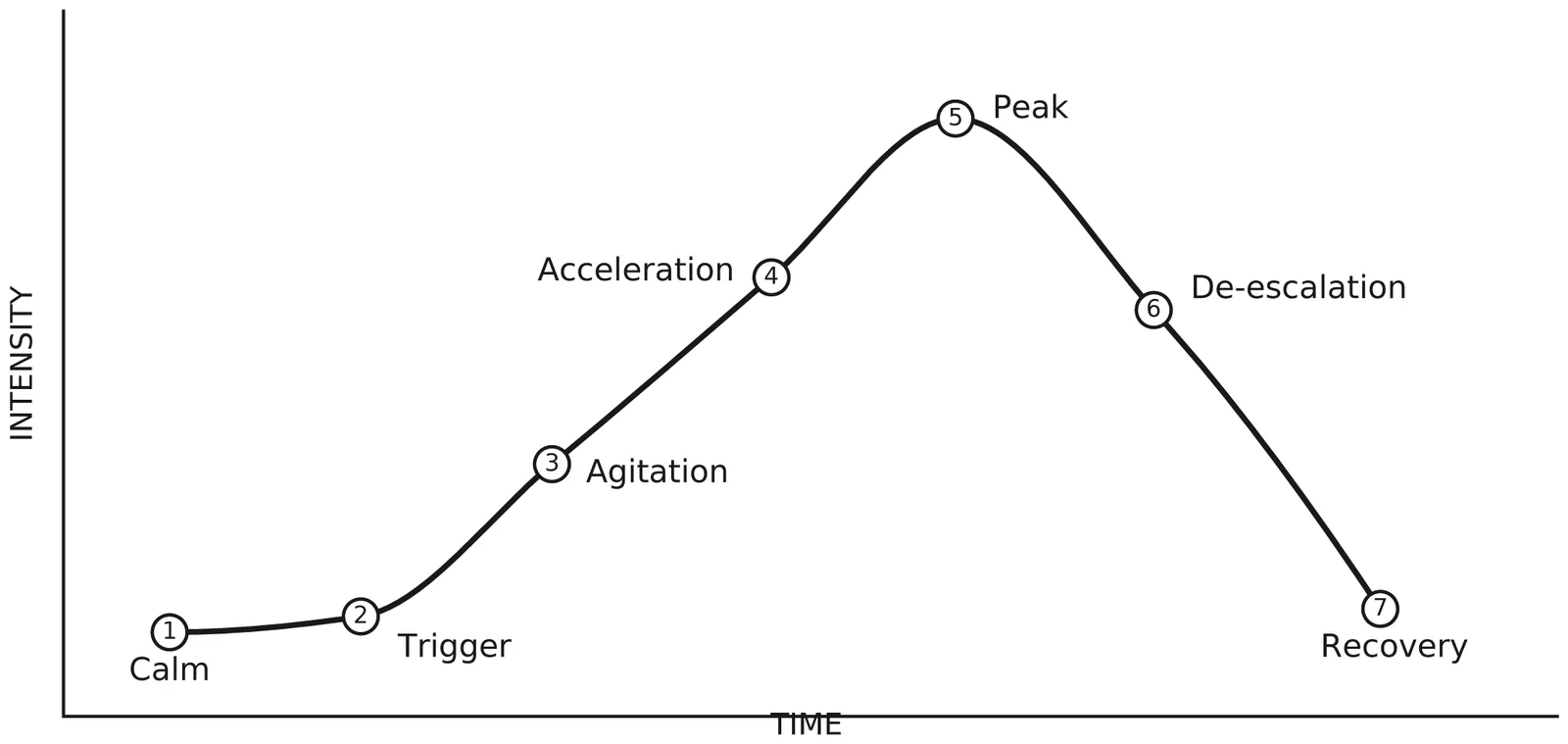
Generic escalation cycle example.

Traditional approaches to managing maladaptive behaviors have relied heavily on behavioral observation, functional behavior assessments, and structured interventions rooted in Applied Behavior Analysis (ABA) (57). While these methods have demonstrated efficacy in reducing the frequency and severity of challenging behaviors, they are inherently reactive and do not allow for identification of internal states acting as markers of escalation. That is, behavioral approach typically addresses behaviors only after they have been observed, limiting the opportunity for early intervention and prevention and intervention at the Trigger point or Agitation point before Acceleration. This limitation has prompted a growing interest in identifying early indicators such as biomarkers of escalation that can inform more proactive and preventative strategies. One of the most promising developments in this area is the use of psychophysiological monitoring to detect early signs of emotional and behavioral dysregulation. The autonomic nervous system plays a central role in regulating emotional and physiological responses to stress and is therefore highly relevant to understanding behavioral escalation in students with ASD (58). Physiological signals such as HRV, GSR, and ST are increasingly recognized as valuable indicators of ANS activity. These biomarkers offer a non-invasive and tolerable means of capturing real-time data on an individual’s physiological and psychological arousal, providing insights into cognitive and emotional states that may not be externally observable, particularly in individuals with limited verbal communication skills (59). The use of these metrics allows the connection of the ABA framework to the CBT framework.

HRV, in particular, has been widely studied as a biomarker of emotional dysregulation and stress (60). HRV refers to the variation in time intervals between heartbeats and is influenced by the balance between the sympathetic and parasympathetic branches of the ANS. Higher HRV is generally associated with greater emotional flexibility, adaptive emotional regulation, and resilience, whereas lower HRV is commonly associated with stress, anxiety, emotional dysregulation, and reduced capacity for self-regulation (60). In individuals with ASD, atypical HRV patterns have been observed during emotional dysregulation, suggesting a potential biomarker for emotional dysregulation and behavioral risk (61, 62). Similarly, GSR, which measures changes in skin conductance due to sweat gland activity, is a well-established indicator of sympathetic nervous system arousal (63, 64). Increases in GSR have been associated with heightened emotional states, including anxiety and agitation. ST, though less frequently examined, also reflects autonomic activity, with decreases often indicating stress-related vasoconstriction in skin surface blood vessels (65). In addition to physiological signals, movement data captured through accelerometers has been used to monitor behavioral states. Accelerometers measure the intensity and frequency of movement, providing a continuous stream of data that can reflect restlessness, pacing, or other motor behaviors associated with agitation (66). When combined with physiological data, movement data can enhance the accuracy of behavioral predictions by capturing both internal and external manifestations of dysregulation. This multimodal approach aligns with the broader trend in behavioral science toward integrating diverse data sources to develop more comprehensive models of behavior.

Modern wearable devices are capable of collecting high-resolution data are lightweight, non-invasive, and can be comfortably worn by children throughout the school day, requiring less tolerance training (67). Algorithms such as Random Forests, Support Vector Machines (SVM), and Long Short-Term Memory (LSTM) networks have been applied to time-series data to identify patterns associated with stress, anxiety, and behavioral outbursts (21, 68). These models are capable of detecting subtle, non-linear relationships among variables and can be trained to recognize escalation patterns specific to individual students helping to personalize interventions (69).

The integration of the ABA and CBT perspectives supports a more holistic understanding of escalation, recognizing that both observable behaviors and internal states contribute to the escalation cycle (70). Despite the promise of these approaches, several challenges remain. One of the primary concerns is the variability in physiological responses among individuals with ASD. Factors such as sensory sensitivities, co-occurring conditions, and medication use can influence baseline physiological states and reactivity, complicating the interpretation of biomarker data. Additionally, the contextual factors that contribute to behavioral escalation such as classroom environment, peer interactions, and instructional demands must be considered when developing predictive models. These factors underscore the importance of collecting data in naturalistic settings and incorporating contextual variables into model training and validation. A second challenge is the ethical and practical considerations associated with continuous physiological monitoring. Issues related to privacy, data security, and informed consent must be addressed, particularly when working with vulnerable populations such as children with disabilities. Furthermore, the implementation of monitoring systems in schools requires collaboration with educators, administrators, and families to ensure that the technology is used in a supportive and non-intrusive manner. Research on the acceptability and usability of wearable devices in educational settings is therefore essential to the successful translation of this technology into practice. In addition to these challenges, there is a need for research which examines the temporal dynamics of behavioral escalation. Understanding how far in advance physiological changes can reliably predict escalation is critical for designing effective intervention protocols. If escalation can be predicted several minutes in advance, educators and support staff may have sufficient time to implement de-escalation strategies. Determining the optimal prediction window is therefore a key focus of current research and has significant implications for the design and implementation of predictive systems.

## Methods

Prior to inclusion in the study participants were screened based upon the following criteria: (1) formal diagnosis of ASD, diagnostic code 299.00 (F84.0); (2) aged 6 to 14; (3) history of behavioral escalation; (4) tolerance for wearing biometric devices; (5) parental consent and participant assent. Potential participants were excluded if there was: (1) the presence of a comorbid, medical, or behavior condition that would interfere with data collection e.g., cardiac conditions or motor control disorders; or (2) lack of informed consent. This approach aligns with an emphasis on using psychophysiological data to understand the cognitive and emotional underpinnings of behavior in real-world contexts (71). Autism diagnoses were confirmed through review of school special education eligibility documentation and existing clinical records provided by parents or guardians. Diagnostic severity levels were assigned according to DSM-5 classifications documented within educational or clinical records. Verbal ability classifications were determined by school speech-language assessments and educational documentation and were categorized as non-verbal, limited verbal, moderate verbal, or fluent verbal communication. The sample ranged in age from 6 to 14 years (M = 10.2 years, SD = 2.1). A total of 37 students were initially screened for participation. Four students did not meet the inclusion criteria and three declined participation or were unable to tolerate the monitoring procedures. Thirty students met all eligibility requirements, provided informed assent and parental consent, and completed the data collection period. No participants withdrew after enrollment (**Table 1**). Of the enrolled participants, 26 demonstrated full tolerance for the wearable sensor throughout the monitoring period, while four required periodic adjustments or brief removal periods resulting in partial data loss. To develop this framework, the study employed an observational design in four rural Northeastern United States public elementary schools, involving 30 students with ASD. Prior to participation, signed informed consent was obtained from parents or legal guardians, and assent was obtained from the students, in accordance with ethical research standards for working with minors and individuals with disabilities.

**Table 1:** Study demographic data.

Demographic category	Frequency
Gender	Male: 16
	Female: 14
Diagnosis Level	Level 2: 15
	Level 1: 11
	Level 3: 4
Verbal Ability	Moderate: 14
	Fluent: 11
	Limited: 4
	Non-verbal: 1
Behavioral Escalation Frequency	High: 12
	Moderate: 11
	Low: 7
Device Tolerance	Yes: 26
	Partial: 4

### Data collection

To mitigate the risk of stigmatization and over-surveillance, the proposed physiological monitoring system is designed with a strong emphasis on privacy, and student autonomy. First, data collection is conducted with informed consent from parents and assent from students, ensuring voluntary participation. The system operates passively and unobtrusively, using wearable devices that are discreet and integrated into the student’s daily routine without drawing attention. Importantly, the system does not label or categorize students based on their physiological data; instead, it provides real-time, anonymized data to educators that indicates the potential pre-escalation states without disclosing sensitive biometric details. All physiological data were encrypted during storage, accessible only to authorized study personnel, and de-identified prior to analysis. These alerts are intended to support not replace professional judgment, helping educators to respond using individualize strategies rather than punitive measures. Furthermore, the system is designed to be inclusive and adaptive, recognizing the diversity of physiological baselines among students with ASD and avoiding one-size-fits-all interpretations. By focusing on early support rather than behavioral correction, the system promotes a proactive, strengths-based approach that aligns with inclusive education principles and fosters a supportive learning environment.

Over a four-week period, biometric and behavioral data were collected during regular school hours from 9:00 AM to 3:30 PM, embedded within the natural rhythms of the classroom environment. Each student wore a small biometric device on one finger the BIOPAC Research Ring during the school day over a four-week period. The device was applied each morning by trained research staff and removed at the end of the school day, with checks to ensure data integrity and participant comfort. The BIOPAC Research Ring is a wearable sensor device used to collect high-fidelity physiological data in ecologically valid settings such as classrooms, clinics, or community settings. The ring is a specifically designed to function as a compact, unobtrusive sensor enabling continuous, non-invasive monitoring of autonomic and behavioral indicators. It captures electrodermal activity (EDA) (32 Hz) also known as galvanic skin response (GSR), a proxy for sympathetic nervous system arousal. Additionally, a photoplethysmography (PPG) is used to measure heart rate variability (HRV) (64 Hz), and pulse which are used as indicators of stress and anxiety. It also monitors peripheral skin temperature (ST) (1 Hz). Lastly the sensor includes a tri-axial accelerometer (100 Hz) to detect movement and activity levels which are indicators of agitation or restlessness. When a secondary point of contact is established, the device is also capable of recording brief electrocardiogram (ECG) signals though due to the more invasive nature of ECG data collection this feature was not used. These signals were selected for their established relevance to autonomic nervous system activity and behavioral arousal, offering a multimodal lens through which to observe the escalation process. Raw signal data from the sensor was collected and processed using AcqKnowledge software, allowing for real-time data acquisition and analysis. The small size and integration capabilities make it well-suited for psychophysiological and behavioral research in naturalistic environments, such as classrooms or clinical settings, where traditional laboratory instrumentation may be impractical or disruptive.

Behavioral incidents were logged by trained members of the research team using a standardized rubric that categorized escalation events by severity and context. Examples of logged behaviors included low-level agitation, such as fidgeting, repetitive movements (e.g., hand-flapping, rocking), increased vocalizations, and mild avoidance or non-compliance. Moderate escalation behaviors included verbal outbursts, pacing, task refusal with emotional intensity, and intensified self-stimulatory behaviors. At the highest level, crisis behaviors included aggression toward others (e.g., hitting, kicking), self-injurious actions (e.g., head-banging, biting), property destruction, and elopement attempts. Each behavioral event was timestamped and later synchronized with the physiological data to enable temporal alignment. Behavioral observation tablets were synchronized daily with the physiological recording system using network time protocols. Event timestamps were recorded to the nearest second and aligned with physiological data streams using a common system clock. Physiological windows occurring within five minutes before an observed escalation event were classified as pre-escalation periods. To ensure consistency and reliability in behavioral coding, interrater reliability was assessed using Cohen’s kappa, with a subset of 20% of the observations was independently coded by two raters. The resulting kappa coefficient was 0.87, indicating strong agreement and high reliability in the application of the rubric (72). This approach allowed for the identification of physiological patterns that preceded observable behavioral escalation, forming the foundation for subsequent predictive modeling. The data collection was embedded within the natural rhythms of the school day, ensuring ecological validity and minimizing disruption to students’ routines. Any discrepancies between coders were resolved through consensus review prior to synchronization with physiological data and subsequent model training. This procedure reduced the likelihood of labeling inconsistencies influencing model performance estimates.

### Data analysis

The analysis of the collected data followed a multi-step process designed to align the multiple heterogeneous physiological signals with the observed behavioral events and extract features specifically for escalation identification and predictive modeling. The primary objective was to identify physiological patterns within HRV, EDA, ST, and movement that reliably preceded behavioral escalation in students with ASD. To accomplish this, initially a time-series analysis was conducted to synchronize the physiological data streams of HRV, GSR, ST, and accelerometer data with the behavioral event logs identified through observation and timestamp. Each behavioral incident was timestamped during data collection, allowing for temporal alignment with the corresponding physiological data. This alignment enabled the identification of physiological changes occurring in the minutes leading up to each escalation event.

Following synchronization, a feature extraction process was applied to the physiological and movement data. For HRV, both time-domain (e.g., RMSSD, SDNN) and frequency-domain features (e.g., low-frequency.04Hz -.15 Hz and high-frequency bands.15 Hz-.4 Hz) were computed to capture autonomic nervous system HRV response dynamics (73). Low-frequency bands are associated with both sympathetic and parasympathetic activity which high-frequency bands primarily reflect parasympathetic activity (20, 74). The ratio of high-frequency to low-frequency is used as an index of autonomic balance i.e., higher ratios indicate stress and arousal which lower ratios indicate relaxation and recovery. HRV is a well-established biomarker of emotional regulation and stress, reflecting the balance between sympathetic and parasympathetic nervous system inputs. From the inter-beat interval (IBI) data, both time-domain and frequency-domain features were extracted. Time-domain features included the Root Mean Square of Successive Differences (RMSSD), which captures short-term variability in heart rate and is sensitive to parasympathetic activity, and the Standard Deviation of NN intervals (SDNN), which reflects overall variability and autonomic flexibility (75). Frequency-domain features were computed using Fast Fourier Transform (FFT) and included low-frequency (LF) power (0.04–0.15 Hz), high-frequency (HF) power (0.15–0.4 Hz), and the LF/HF ratio. These features provide insights into the student’s physiological state, with elevated LF/HF ratios often associated with stress or arousal.

Galvanic skin response (GSR), is a direct measure of sympathetic nervous system activation, was analyzed to detect changes in electrodermal activity (76). GSR is particularly sensitive to emotional arousal and has been shown to increase in anticipation of or during stressful events. Galvanic Skin Response (GSR) data were analyzed to quantify sympathetic nervous system activity through the extraction of key temporal features associated with electrodermal responses. Prior to feature extraction, the raw GSR signals were preprocessed using standard filtering techniques to reduce noise and eliminate motion artifacts. The raw GSR signals were preprocessed using a zero-phase Butterworth low-pass filter to enhance signal quality and reduce noise artifacts (77). This involved applying standard filtering techniques, with a 1 Hz cutoff to remove high-frequency noise and baseline correction of.01 Hz to account for slow signal drifts. These preprocessing steps ensured that the extracted features accurately reflected physiological responses rather than artifacts introduced by movement or environmental interference. Event-related skin conductance responses (SCRs) were identified using a threshold-based detection algorithm, ensuring that only physiologically relevant responses were included in the analysis (78). This method involved identifying local maxima in the signal that exceed a predefined amplitude threshold, typically set relative to the baseline or noise level of the signal. In this study, Skin conductance responses (SCRs) were defined as conductance increases exceeding 0.01–0.05 µS, with an onset-to-peak rise occurring within a window of 1–5 seconds, consistent with established psychophysiological criteria. The threshold was selected to minimize false positives due to noise while ensuring sensitivity to genuine autonomic responses. Detected SCRs were then used for subsequent feature extraction, including peak amplitude, rise time, and recovery time. Peak amplitude was defined as the maximum increase in skin conductance relative to the pre-stimulus baseline, serving as an index of arousal intensity. Rise time was measured as the interval between the onset of the SCR and its peak, reflecting the sympathetic response. Recovery time captured the duration required for the skin conductance to return from peak to baseline levels, providing insight into the autonomic system’s return to homeostasis. These features collectively offer a robust characterization of sympathetic arousal in response to experimental stimuli. Additionally, the number of discrete peaks within each time window was counted to quantify the frequency of arousal events. These features collectively provide a view of the student’s physiological reactivity and recovery patterns.

Skin temperature, recorded via thermistors embedded in the wearable device, was analyzed to detect stress-related changes in peripheral blood flow (79). Under stress, vasoconstriction can lead to a measurable drop in skin temperature, particularly in the extremities. For each time window, the mean skin temperature was calculated, along with the slope of temperature change and the deviation from the individual’s baseline (80). These features were used to identify subtle thermal shifts that may precede behavioral escalation, offering an additional physiological dimension to the predictive model. Skin temperature data were processed to identify deviations from baseline and rate of change, while accelerometer data were used to compute movement intensity, variance, and entropy. These features were extracted using sliding windows of fixed duration (e.g., 30 seconds) to preserve temporal resolution. To prepare the data for machine learning, each time window was labeled based on its temporal proximity to a behavioral escalation event. Windows occurring within a defined pre-escalation period (e.g., 5 minutes before an incident) were labeled as “pre-escalation, “ while windows from calm periods were labeled as “non-escalation.” This binary classification scheme enabled the training of supervised learning models to distinguish between physiological states associated with imminent escalation and those reflecting baseline or regulated behavior. This analytical framework provided the foundation for subsequent model training and evaluation, allowing for the development of predictive algorithms capable of identifying early warning signs of behavioral escalation.

Accelerometer data were processed to capture movement patterns associated with agitation, restlessness, or physical escalation. The three-axis (X, Y, Z) accelerometer provided continuous data on movement intensity and direction. From this signal, several features were extracted to create the activity index (computed as the vector magnitude of acceleration across all three axes), standard deviation (to assess variability in movement), jerk (the rate of change in acceleration, capturing abrupt movements), and entropy (a measure of movement complexity and unpredictability). These features were particularly useful for identifying physical manifestations of dysregulation that may not yet be behaviorally classified as escalation but indicate a shift in arousal or engagement (81). All features were extracted using a sliding window approach, with 30-second windows and 50% overlap to ensure sufficient temporal resolution while minimizing data loss (82). This windowing strategy allowed for the continuous monitoring of physiological and movement states and enabled the detection of gradual changes over time. Each window was treated as a discrete observation and was labeled based on its temporal proximity to a documented behavioral escalation event. Windows occurring within a five-minute period prior to an escalation were labeled as “pre-escalation, “ while those occurring during calm periods were labeled as “non-escalation.” This binary classification scheme provided the foundation for supervised machine learning, allowing models to learn the physiological signatures associated with impending behavioral events.

To transform raw physiological and movement data into a structured format suitable for predictive modeling, a comprehensive feature extraction process was implemented. This process was designed to capture the dynamic physiological and behavioral states of students with ASD in the moments leading up to behavioral escalation. By extracting meaningful features from continuous data streams, the study aimed to identify patterns that could serve as early indicators of dysregulation. The feature extraction procedure was applied to four primary data modalities collected via the sensor, HRV, GSR, ST, and three-axis accelerometer data. Each signal was processed within fixed-length, overlapping time windows to preserve temporal continuity and enhance the resolution of physiological changes over time. To ensure the robustness of the extracted features, preprocessing steps as described were applied to each signal prior to feature computation. HRV data were filtered to remove motion artifacts and ectopic beats, and IBIs were interpolated to create evenly spaced time series. GSR signals were smoothed using a low-pass filter to reduce noise and baseline drift. Skin temperature data were adjusted for ambient environmental conditions using classroom temperature logs, and accelerometer data were normalized to account for differences in device orientation and placement. These preprocessing steps were essential for enhancing signal quality and ensuring the reliability of the extracted features.

To ensure the integrity of the physiological and movement data collected via wearable sensors, a comprehensive preprocessing pipeline was implemented to address common challenges such as signal noise, sensor dropout, and missing data. Raw signals were first subjected to modality-specific filtering techniques: HRV data were processed using bandpass filters to isolate cardiac frequencies and remove motion artifacts; GSR signals were smoothed using a zero-phase Butterworth low-pass filter with baseline correction to eliminate drift and high-frequency noise; and accelerometer data were normalized to account for device orientation and environmental variability. Missing data segments, typically resulting from brief sensor disconnections or poor skin contact, were identified through signal continuity checks and interpolated using linear or spline methods when gaps were under 5 seconds. Longer gaps were excluded from analysis to preserve data validity. To enhance temporal resolution and capture dynamic physiological changes, a sliding window approach was employed with 30-second windows and 50% overlap. This window size was selected based on prior literature in psychophysiological monitoring, which suggests that 30-second epochs are optimal for capturing short-term autonomic fluctuations while maintaining computational efficiency (e.g., Nayak et al., 2023; Pernice et al., 2019). The 5-minute pre-escalation labeling window was determined through exploratory analysis of behavioral logs and physiological trends, which consistently indicated that meaningful changes in HRV and GSR occurred within this timeframe prior to observable behavioral escalation. This labeling strategy was further supported by existing studies in affective computing and behavioral prediction, which have identified similar lead times as critical for anticipatory intervention (e.g., Khan et al., 2024; Popescu et al., 2025). Together, these preprocessing and labeling decisions ensured that the machine learning models were trained on high-quality, temporally aligned data capable of capturing the nuanced physiological signatures associated with behavioral dysregulation. Total data loss during processing for each measure was: HRV 6.2%, GSR 9.2%, ST: 7.1%, and accelerometer data: 7.7%.

### Machine learning model training

The dataset used for model training consisted of labeled feature vectors extracted from synchronized physiological and behavioral data. Each vector represented a 30-second time window and was labeled as either “pre-escalation” or “non-escalation” based on its temporal proximity to a documented behavioral incident. To ensure balanced representation and prevent overfitting, the dataset was randomly split into training (70%) and testing (30%) subsets using stratified sampling to maintain class distribution. The training set was used to fit the models, while the testing set was reserved for evaluating generalization performance. The raw data were processed and transformed into feature vectors representing physiological and movement patterns. These vectors were labeled based on their temporal proximity to documented behavioral escalation events, allowing for the identification of pre-escalation physiological signatures i.e., biomarkers. Machine learning models including Random Forests, Support Vector Machines, and Long Short-Term Memory (LSTM) networks were trained to classify these patterns and predict escalation events (83). The models were evaluated using cross-validation and performance metrics i.e., accuracy, precision, recall, and F1-score (84).

To evaluate the predictive capacity of physiological and movement data in identifying behavioral escalation in students with ASD a supervised machine learning framework was implemented. The objective was to train models capable of distinguishing between physiological states associated with pre-escalation and those reflecting non-escalation or baseline conditions. This approach allows for the development of a sensor driven system that could potentially alert educators to early signs of dysregulation, enabling timely and targeted interventions. Essentially, this approach enabled the identification of discriminative physiological patterns associated with specific behavioral states, providing insights into autonomic and behavioral regulation in students with ASD. Three machine learning models were selected for this study: Random Forest, Support Vector Machine (SVM), and Long Short-Term Memory (LSTM) neural networks. These models were chosen for their complementary strengths in handling high-dimensional, time-series data such as in this study. The Random Forest classifier is an ensemble learning method based on decision trees, was selected for its robustness to noise, interpretability, and ability to model non-linear relationships (85). The SVM model was implemented with a radial basis function (RBF) kernel, was used for its effectiveness in separating complex feature spaces, particularly in cases where the boundary between escalation and non-escalation states may not be linearly separable (86). The RBF kernel maps input features into a higher-dimensional space, allowing the model to construct non-linear decision boundaries that better capture the complex physiological and behavioral patterns present in the data. Hyperparameters, including the regularization parameter C and the kernel coefficient γ, were optimized using grid search with cross-validation to prevent overfitting and ensure generalizability. The RBF kernel was particularly well-suited for this application, as it enabled the model to distinguish subtle variations in autonomic and behavioral responses among students with ASD. The final model implemented was a Long Short-Term Memory (LSTM) network, a type of recurrent neural network (RNN) designed to capture long-range temporal dependencies in sequential data (87). This architecture was particularly well-suited for modeling physiological signals, where the timing and order of events carry critical information about autonomic and behavioral states. The LSTM model processes input sequences by maintaining a memory cell that selectively retains or forgets information over time, enabling it to learn complex temporal patterns that traditional feedforward networks cannot capture. In this study, the LSTM was trained on time-series data derived from synchronized physiological signals, including heart rate variability, galvanic skin response, and skin temperature. The LSTM model was trained using a sequence of time-ordered feature vectors, with tuning focused on the number of hidden units, dropout rate, and learning rate. The model was optimized using backpropagation through time (BPTT), and performance was evaluated using standard classification metrics (88). This approach allowed for the dynamic modeling of physiological responses, providing a robust framework for detecting state changes in students with ASD.

## Results

The results of this study provide evidence for the predictive utility of physiological and movement data in identifying behavioral escalation in students with ASD, offering a transformative approach to proactive behavioral intervention in educational settings. Through the application of machine learning techniques to multimodal biometric data collected in real-time from wearable sensors, the study demonstrated that internal physiological states—specifically heart rate variability (HRV), galvanic skin response (GSR), skin temperature, and accelerometer-derived movement patterns can serve as reliable indicators of impending behavioral dysregulation. Across all observations, a total of 200 behavioral events were recorded. **Table 2** presents both the frequency and percentage of each behavior type.

**Table 2:** Distribution of observed behavioral events.

Behavior type	Percentage	Frequency	Example behaviors
Mild Agitation	24	48	Fidgeting, shifting in seat, facial tension, sighing, disengagement from task
Repetitive Behaviors	18	36	Hand-flapping, rocking, finger flicking, repetitive vocal sounds
Increased Vocalization	14	28	Repeated questioning, louder speaking volume, vocal perseveration, humming
Task Refusal	12	24	Turning away from work, putting head down, saying “no, “ refusing instructions
Verbal Outburst	10	20	Yelling, crying, screaming, verbal threats, profanity
Aggression	9	18	Hitting, kicking, biting, pushing, throwing objects at others
Self-Injury	8	16	Head banging, self-hitting, scratching self, biting self
Elopement	5	10	Running from classroom, leaving assigned area, bolting from staff
Total	100	200	

Across all documented behavioral incidents, mild agitation (24%) and repetitive behaviors (18%) were the most frequently observed antecedent behaviors. Higher-severity events such as aggression (9%), self-injury (8%), and elopement (5%) occurred less frequently but represented the majority of incidents requiring immediate staff intervention. These findings suggest that behavioral escalation was typically characterized by a progression from low-intensity dysregulation to more severe behavioral manifestations.

Descriptive statistics demonstrated systematic physiological differences between pre-escalation and non-escalation periods. Pre-escalation windows were associated with lower HRV values (RMSSD: M = 24.8 ms, SD = 7.2) relative to non-escalation windows (RMSSD: M = 36.5 ms, SD = 8.9). Similarly, the LF/HF ratio was elevated during pre-escalation periods (M = 2.84, SD = 0.91) compared to non-escalation periods (M = 1.61, SD = 0.73), indicating increased sympathetic activation. GSR activity demonstrated greater peak amplitude during pre-escalation periods (M = 0.22 µS, SD = 0.08) compared to non-escalation periods (M = 0.11 µS, SD = 0.05). Skin temperature demonstrated modest reductions during pre-escalation periods (M = 30.8 °C, SD = 0.7) relative to non-escalation periods (M = 31.3 °C, SD = 0.6). Accelerometer-derived movement variability was also elevated during pre-escalation periods (M = 1.48 g, SD = 0.39) compared with non-escalation periods (M = 0.91 g, SD = 0.27). Collectively, these findings are consistent with increased autonomic arousal and behavioral activation preceding observed escalation events.

Among the three machine learning models evaluated—Random Forest (RF), Support Vector Machine (SVM), and Long Short-Term Memory (LSTM) neural networks the LSTM model emerged as the most effective, achieving an overall classification accuracy of 85.2% in distinguishing between “pre-escalation” and “non-escalation” states. Importantly, all analyses reported in this study were conducted retrospectively using previously collected and synchronized physiological and behavioral data. The predictive system was not deployed in real time during data collection. Rather, model performance reflects *post hoc* classification of pre-escalation states and should therefore be interpreted as evidence of feasibility rather than evidence of operational effectiveness in live educational settings. This performance was validated using a held-out test set comprising 30% of the total dataset, with stratified sampling employed to preserve class balance and ensure robust generalization. The LSTM model also achieved a precision of 83.7%, a recall of 86.5%, and an F1-score of 85.1%, indicating a well-balanced ability to detect true positives while minimizing false positives and false negatives. These metrics were further supported by an area under the receiver operating characteristic curve (AUC) of 0.90, reflecting excellent discriminative capacity across a range of decision thresholds. In comparison, the Random Forest model achieved an accuracy of 81.4%, precision of 79.2%, recall of 82.6%, F1-score of 80.9%, and AUC of 0.88, while the SVM model yielded slightly lower performance with an accuracy of 78.9%, precision of 76.4%, recall of 80.1%, F1-score of 78.2%, and AUC of 0.83. See **Figure 3**. These results underscore the superior performance of the LSTM model, particularly in capturing the temporal dependencies inherent in physiological time-series data. The F1-score, was used as a balanced metric to assess performance in the presence of class imbalance. These metrics were computed on the held-out test set to assess each model’s effectiveness in predicting behavioral escalation from physiological and movement data. The physiological data were segmented into 30-second overlapping windows, from which features such as RMSSD, LF/HF ratio, GSR peak amplitude, and movement entropy were extracted. Each window was labeled as either “pre-escalation” or “non-escalation” based on its proximity to a documented behavioral event. These labeled feature vectors were used to train and evaluate three machine learning models such as a Random Forest, SVM, and LSTM neural networks. The LSTM model, which excelled at capturing temporal dependencies in sequential data, achieved the highest predictive accuracy at ~85%, identifying escalation patterns 2–5 minutes before observable behaviors occurred with reduction in accuracy occurring at a rate of 3% per minute increase in the time before escalation. This model was then integrated into a simulated alert system that processed incoming physiological data, applied the trained model, and triggered alerts when pre-escalation patterns were detected. Alerts were designed to be discreet and informative, providing the student’s anonymized ID, prediction confidence, and potential time to escalation. Future iteration will also provide potential de-escalation strategies for the individual student. Although the system was not deployed live, retrospective testing demonstrated its feasibility and potential for real-world application. Retrospective analysis indicated an estimated false-positive rate of approximately 0.18 alerts per student-hour when prediction confidence thresholds were optimized for recall. Although not evaluated under operational conditions, these findings suggest that alert frequency may remain manageable for educators while preserving sensitivity to imminent escalation events. Future live-deployment studies will be necessary to determine acceptable alert thresholds and their impact on educator workload.

**Figure 3:**
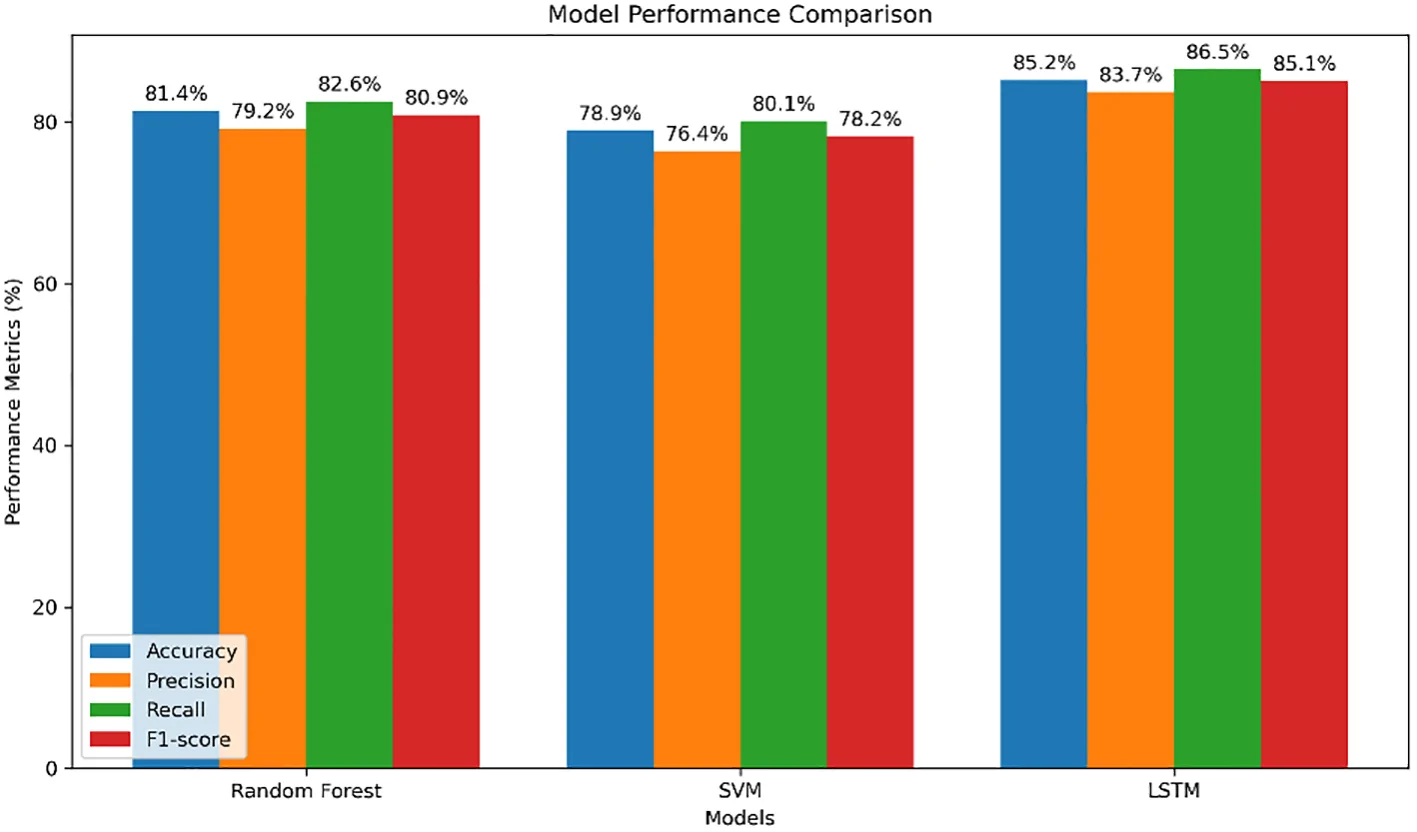
Comparison of model performance metrics (accuracy, precision, recall, and F1-score) for Random Forest, Support Vector Machine (SVM), and Long Short-Term Memory (LSTM) models.

A detailed feature importance analysis revealed that HRV followed by GSR were the most predictive physiological indicators of escalation (**Figure 4**). HRV, is already a well-established biomarker of autonomic nervous system regulation, contributed significantly to model performance through both time-domain features such as the Root Mean Square of Successive Differences (RMSSD) and the Standard Deviation of NN intervals (SDNN), as well as frequency-domain features. These features reflect the balance between sympathetic and parasympathetic nervous system activity and were particularly sensitive to shifts from calm, regulated states to dysregulated, aroused states. GSR, which measures electrodermal activity and is a direct index of sympathetic nervous system arousal, also emerged as a critical though secondary predictor. Features such as peak amplitude, rise time, recovery time, and the frequency of discrete peaks within each time window provided nuanced insights into the intensity and duration of physiological arousal. Notably, the inclusion of GSR features alone resulted in a 12% increase in recall when added to models trained only on HRV and accelerometer data, highlighting its unique contribution to early detection of stress-related physiological changes.

**Figure 4:**
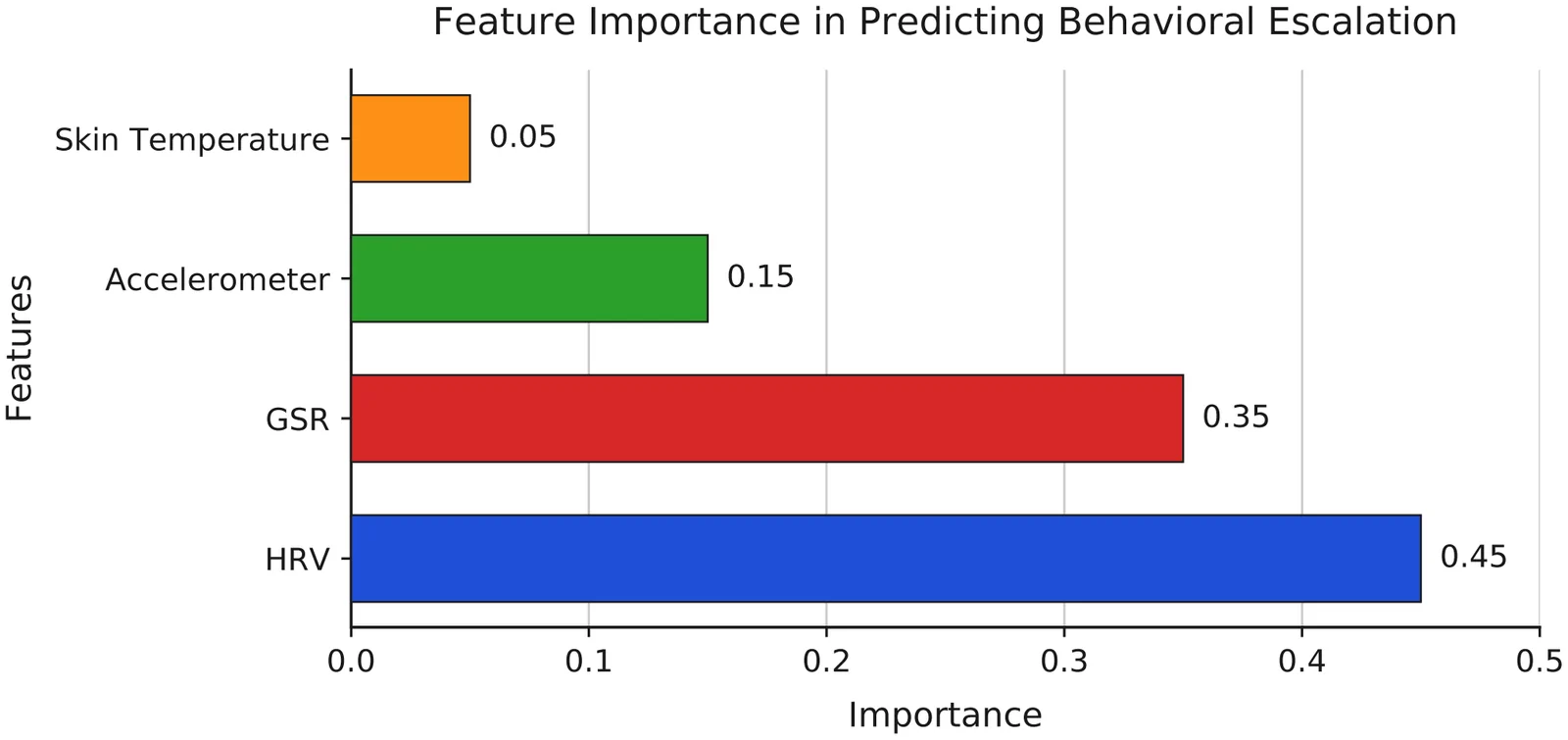
Relative importance of physiological and movement features in predicting behavioral escalation. This horizontal bar chart illustrates the contribution of each feature to the predictive model.

Although skin temperature was not predictive on its own, it contributed to the model’s sensitivity when combined with other features such as HRV and GSR. Stress-induced vasoconstriction, which often results in peripheral temperature drops, was captured through features such as mean temperature, rate of change, and deviation from baseline. These thermal shifts in the extremities, while subtle, provided additional context for interpreting autonomic nervous system activity, particularly in conjunction with HRV and GSR. The accelerometer data, which captured movement intensity, variability, and entropy across three axes, significantly enhanced the model’s predictive power. Movement features such as the activity index, jerk (rate of change in acceleration), and entropy (a measure of movement complexity) were particularly useful in identifying physical manifestations of agitation that often precede overt behavioral escalation. When accelerometer data were integrated with physiological features, the LSTM model’s F1-score improved by 3.3 percentage points, confirming the hypothesis that a multimodal approach yields higher predictive accuracy than any single modality. To further validate the effect of combining data modalities, neural network ablation studies were conducted to assess the impact of removing individual feature sets. Models trained on HRV and GSR alone achieved an F1-score of 81.2%, while the addition of accelerometer data increased the score to 85.1%. Including all four modalities HRV, GSR, skin temperature, and accelerometer resulted in a marginal improvement to 85.2%, suggesting that while skin temperature adds value, the core predictive power lies in the integration of HRV, GSR, and movement data. These findings support the study’s second hypothesis, which posited that a multimodal feature set combining HRV and GSR would yield higher predictive accuracy than any single physiological or movement feature alone. The LSTM model outperformed the other classifiers across all metrics, achieving the highest accuracy (85.2%), recall (86.5%), and F1-score (85.1%), indicating its superior ability to detect pre-escalation states in students with ASD. This visualization highlights the effectiveness of temporal modeling in capturing physiological patterns associated with behavioral dysregulation.

One of the most critical findings of the study was the model’s ability to predict behavioral escalation 2 to 5 minutes before the onset of observable behaviors. This temporal prediction window was determined by aligning the model’s predicted pre-escalation labels with the actual timestamps of documented behavioral incidents. The LSTM model maintained a true positive rate of ~86.5% within this window, providing a meaningful opportunity for educators and support staff to implement proactive interventions. This lead time is pedagogically significant, as it allows for the deployment of de-escalation strategies such as sensory breaks, environmental modifications, or calming techniques before a student reaches a crisis point. The ability to anticipate escalation several minutes in advance represents a substantial improvement over traditional behavioral observation methods, which are inherently reactive and often fail to capture the internal physiological states that precede dysregulation. **Figure 5** illustrates the dynamic changes in physiological markers—heart rate variability (HRV) and galvanic skin response (GSR) during the 10-minute period leading up to a documented behavioral escalation event in a student with ASD. The data demonstrate a clear inflection point approximately five minutes prior to escalation, where HRV begins to decline and GSR begins to rise. The drop in HRV reflects a reduction in parasympathetic activity and autonomic flexibility, commonly associated with increased emotional stress and diminished self-regulatory capacity. Concurrently, the spike in GSR indicates heightened sympathetic nervous system arousal, a physiological correlate of emotional agitation and cognitive overload. These trends align with established psychophysiological models of emotional dysregulation and support the use of HRV and GSR as early biomarkers of behavioral risk. The visualization underscores the practical utility of a 2–5-minute prediction window, during which educators and support staff can implement proactive, individualized interventions to prevent escalation. By capturing these subtle yet significant physiological shifts, the system offers a data-driven foundation for anticipatory behavioral support, bridging the gap between internal emotional states and observable behaviors.

**Figure 5:**
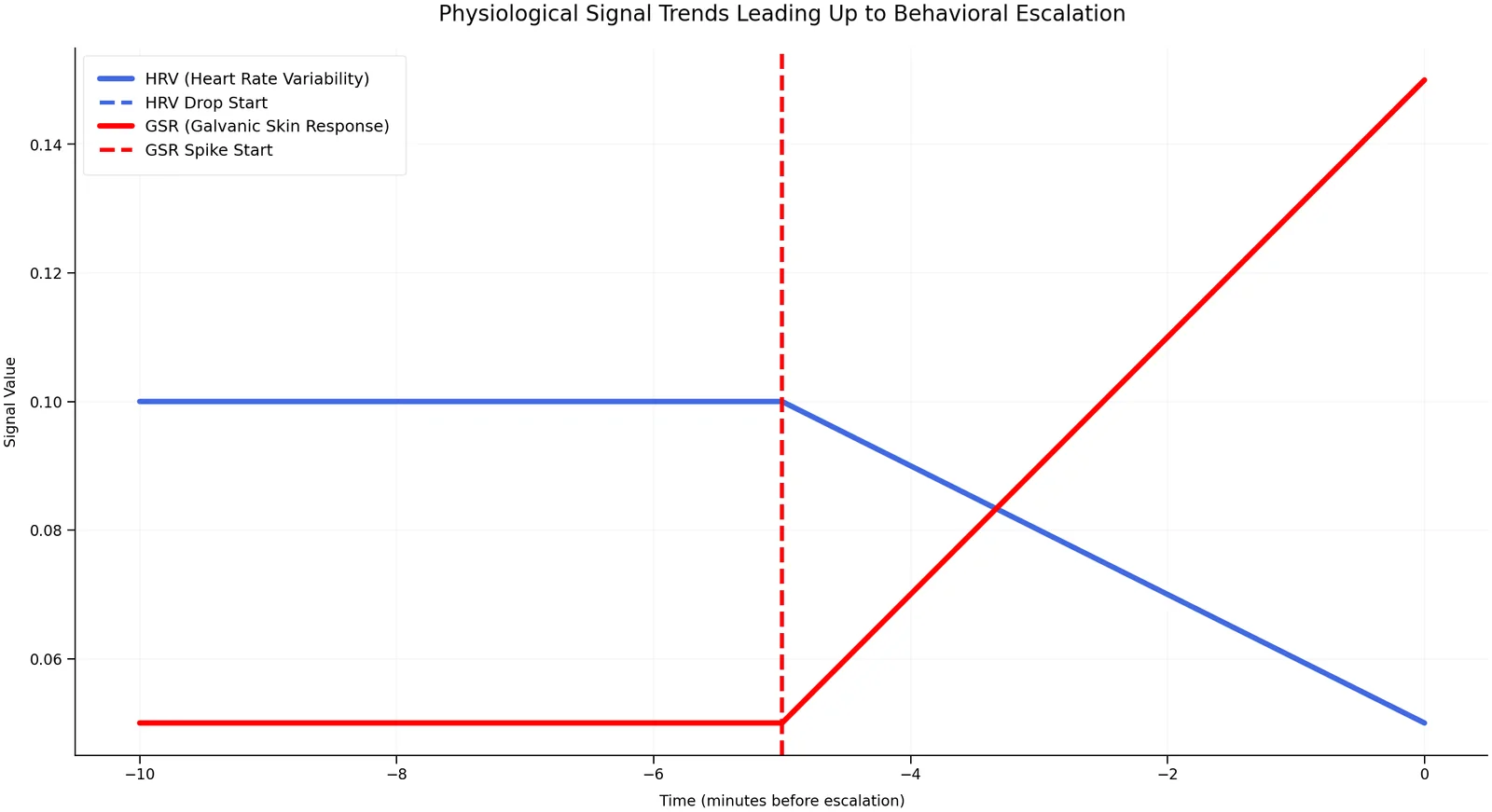
Temporal prediction window.

To assess the generalizability of the model across individuals, a leave-one-subject-out cross-validation (LOSO-CV) analysis was performed (89). In this approach, the model was trained on data from all but one participant and tested on the excluded participant, with the process repeated for each student. The LSTM model maintained an average accuracy of ~82.6% across all folds, demonstrating its robustness in predicting escalation for students not included in the training data. This finding is particularly important for real-world deployment, where predictive systems must generalize to new students with diverse physiological baselines, behavioral profiles, and contextual factors. The model’s ability to maintain high performance across individuals supports its scalability and of practical utility in inclusive educational environments. In addition to quantitative performance metrics, qualitative observations from the behavioral logs provided further validation of the model’s predictions. In several instances, the model correctly identified pre-escalation states during periods of subtle agitation such as increased fidgeting, repetitive movements, or vocalizations that were not initially classified as escalation by human observers but were later confirmed as precursors to more intense behaviors. These cases suggest that the model may be capable of detecting early physiological or biomarker signals that are not readily observable, offering a valuable complement to traditional behavioral observation methods. This capability is particularly relevant for students with limited verbal communication skills, who may struggle to express internal distress through conventional means.

## Discussion

The results of this study support previous studies connecting the use of ANS signals for the monitoring of behavioral escalation (38, 59, 90). Importantly, the model’s generalizability across participants, as demonstrated by the leave-one-subject-out cross-validation, indicates that this approach can be scaled and adapted to diverse student populations. The ability to maintain high predictive accuracy across individuals with varying physiological baselines and behavioral profiles is essential for real-world implementation in educational settings. Nevertheless, predictive performance was evaluated under retrospective conditions and should not be interpreted as evidence that comparable accuracy will be achieved during live implementation. Prospective validation studies conducted across multiple schools, classroom contexts, and educator teams will be necessary to determine operational effectiveness under naturally occurring educational conditions. Moreover, the successful simulation of a prototype alert system illustrates the translational potential of this research. By providing educators with timely, objective insights into students’ physiological states, such systems can shift the paradigm from reactive behavior management to anticipatory, individualized support an approach consistent with both CBT’s emphasis on proactive coping strategies and ABA’s focus on antecedent-based interventions. This approach also addresses several limitations of traditional behavioral observation methods, which are often subjective, inconsistent, and delayed. Physiological monitoring using biomarkers offers a continuous, non-invasive, and data-driven alternative that can detect internal states not readily observable, particularly in students with limited verbal communication skills. The integration of machine learning further enhances this capability by identifying complex, non-linear patterns in high-dimensional data that would be difficult to discern through manual analysis. Ultimately, this study demonstrates that biometric data can be effectively used to predict behavioral escalation in students with autism, offering a novel and impactful tool for enhancing classroom management and individualized support. By enabling early detection and intervention, this approach has the potential to reduce the frequency and severity of behavioral incidents, improve student well-being, and foster more inclusive and supportive learning environments.

### Application to practice

This study represents an advancement in the intersection of behavioral science, cognitive psychology, and educational technology by operationalizing emotional dysregulation through real-time physiological and movement data in students with ASD. A structured intervention protocol should accompany system alerts to ensure consistent implementation. For example, low-risk alerts may prompt increased observation and informal student check-ins, moderate-risk alerts may initiate environmental modifications or sensory regulation strategies, and high-risk alerts may warrant individualized de-escalation procedures identified within the student’s behavioral support plan. The effectiveness of these interventions could be evaluated through reductions in escalation frequency, decreases in behavior severity, shorter recovery periods, and improvements in classroom participation. Establishing a standardized response framework will be important for future implementation studies. Traditional psychological theories of emotional regulation, particularly those grounded in Cognitive Behavioral Therapy (CBT), rely heavily on introspective self-report and verbal articulation of internal states—methods that are often inaccessible to individuals with limited communication abilities. By leveraging biometric indicators such as heart rate variability (HRV), galvanic skin response (GSR), and accelerometer-derived movement patterns, this research challenges the assumption that emotional states must be inferred indirectly or *post hoc*. Instead, it offers a quantifiable, continuous, and objective framework for identifying pre-escalation states, thereby externalizing what CBT conceptualizes as “private events.” Simultaneously, the study extends the Applied Behavior Analysis (ABA) model by introducing physiological markers as antecedent variables within the traditional antecedent-behavior-consequence (ABC) framework. This innovation enables a more nuanced understanding of behavioral escalation, particularly in cases where observable antecedents are ambiguous or absent. The integration of CBT and ABA through psychophysiological assessment not only bridges two historically distinct paradigms but also supports a more holistic and inclusive approach to behavioral intervention. Furthermore, the use of wearable sensors in naturalistic classroom settings challenges the prevailing notion in psychophysiological research that such assessments must be confined to controlled laboratory environments. This ecological validity enhances the translational potential of the findings, making them directly applicable to real-world educational contexts. The predictive modeling approach, particularly the use of Long Short-Term Memory (LSTM) networks, underscores the dynamic and temporal nature of emotional and behavioral regulation, offering a methodological contribution to the field of psychological assessment. By demonstrating that behavioral escalation can be predicted 2–5 minutes before observable behaviors manifest, the study provides a critical window for proactive intervention—an advancement that redefines the temporal boundaries of behavioral support. Collectively, these contributions challenge the reactive nature of traditional behavioral and psychological practices and advocate for a paradigm shift toward anticipatory, data-driven, and individualized support systems for neurodiverse learners.

To support the emotional and behavioral development of students with ASD, a combined intervention framework integrating Cognitive Behavioral Therapy (CBT) and Applied Behavior Analysis (ABA) was implemented. This hybrid approach leverages the structured, evidence-based strategies of ABA such as reinforcement, prompting, and task analysis to shape adaptive behaviors, while incorporating CBT techniques to address cognitive processes underlying emotional regulation and social understanding. The findings of this study have implications for educational practice, particularly in the development and implementation of proactive behavioral support systems for students with ASD and other neurodevelopmental disorders. One of the most immediate applications is the integration of the predictive model into wearable devices capable of real-time physiological monitoring. These devices, can continuously collect biometric data and transmit this information to a centralized system that analyzes the data using trained machine learning models to provide alerts as needed to educators and others. When the system detects physiological patterns indicative of pre-escalation, it can generate discreet alerts for educators and support staff, providing a critical window of 2 to 5 minutes (future work will seek to extend this time) to implement de-escalation strategies before behaviors become disruptive or harmful. To ensure the successful adoption of such systems, it is essential to provide comprehensive training for educators on how to interpret and respond to these alerts. To minimize alert fatigue and excessive educator burden, alert thresholds should be individualized and adjusted according to student-specific physiological baselines and behavioral histories. Rather than generating alerts for every fluctuation in physiological activity, the system should prioritize high-confidence predictions and suppress redundant notifications occurring within short time intervals. Future iterations should evaluate acceptable false-positive rates, educator perceptions of alert usefulness, and optimal notification frequency. These safeguards are essential to maintaining staff trust in the system and ensuring that alerting mechanisms support rather than distract from classroom instruction. Training should include an understanding of the physiological signals being monitored, the biomarkers, the significance of different alert thresholds, and evidence-based intervention strategies that can be deployed in response to early warning signs as indicated by CBT-ABA. This training should also emphasize the importance of individualized responses, as physiological and behavioral profiles can vary widely among students with ASD. By equipping educators with the knowledge and tools to act on real-time data, schools can move from reactive to proactive behavior management, reducing the frequency and severity of crisis incidents and improving overall student outcomes.

Moreover, the use of real-time physiological monitoring promotes a potentially more holistic and personalized approach to behavior management. Rather than relying solely on observable behaviors, which may be misinterpreted or noticed too late, educators can respond to objective, data-driven indicators of distress. This reduces the reliance on punitive measures and supports the creation of learning environments that are responsive to the unique needs of neurodiverse learners. Over time, the consistent use of such systems may also contribute to a cultural shift within schools, fostering greater empathy, understanding, and collaboration among educators, students, and families. The integration of biometric monitoring and predictive modeling into educational practice offers a powerful tool for enhancing behavioral support for students with autism. By combining real-time data collection with machine learning and grounding interventions in ABA and CBT frameworks, schools can implement proactive, personalized strategies that improve student well-being, reduce crisis incidents, and promote more inclusive and supportive learning environments.

### Limitations

However, while the results are promising, several limitations must be acknowledged. A major limitation is that the predictive system was evaluated retrospectively and was not implemented as a live operational intervention during data collection. Consequently, the study cannot determine whether alerts would improve educator response, reduce escalation frequency, or maintain predictive performance under real-time classroom conditions. The sample size, though sufficient for initial modeling, limits the generalizability of the findings to broader populations. Future studies should evaluate the system under fully operational classroom conditions across multiple schools, geographic regions, and educational settings. Such work will be necessary to determine whether performance observed in retrospective analyses can be maintained during real-time implementation where environmental variability, educator decision-making, and operational constraints may influence system effectiveness. Second, the study was conducted exclusively in school settings, specifically within structured classroom environments. While this context was ideal for ensuring ecological validity and relevance to educational practice, it also means that the findings may not generalize to other environments, such as home, clinical, or community settings. Behavioral patterns and physiological responses may differ significantly across contexts due to variations in environmental stimuli, social dynamics, and activity demands. Therefore, additional studies are needed to explore the applicability of this predictive approach in non-school settings and to assess how contextual factors influence model performance. Third, the accuracy and reliability of the physiological data are inherently dependent on the quality and consistency of the wearable sensors used. Although the sensor in this study provided high-resolution data and was well-tolerated by participants, wearable devices are subject to variability due to factors such as sensor placement, movement artifacts, skin contact, and ambient conditions. Addressing these technical challenges will be critical for ensuring the reliability and scalability of real-time monitoring systems These factors can introduce noise into the data and potentially affect the precision of the predictive models. Future implementations should consider incorporating sensor calibration protocols, redundancy across multiple sensors, and real-time data quality checks to mitigate these issues. Another limitation involves the substantial heterogeneity of autism spectrum disorder. Students with ASD demonstrate considerable variability in physiological regulation, communication profiles, sensory sensitivities, and behavioral presentation. Although leave-one-subject-out cross-validation demonstrated reasonable model generalization across participants, additional work is needed to determine whether model performance remains stable across broader populations differing in age, support needs, co-occurring psychiatric conditions, medication status, and educational placement. Future investigations should evaluate personalized modeling approaches that incorporate individual physiological baselines and adaptive learning algorithms. While the study provides a foundation for the use of biometric data in predicting behavioral escalation, these limitations highlight the need for continued research to enhance the robustness, scalability, and contextual adaptability of the approach. In addition, more work is required in the development of the combined CBT-ABA framework for implementation in the classroom. Additionally, the present design cannot establish causal relationships between physiological markers and behavioral escalation. The identified biomarkers should be interpreted as correlates and predictors of escalation risk rather than direct causes of subsequent behavior.

## Conclusion

This study demonstrates that physiological and movement data are associated with behavioral escalation in students with ASD and may support the future development of predictive monitoring systems. Retrospective analyses indicated that machine learning models could classify physiological patterns associated with behavioral escalation approximately 2–5 minutes before documented behavioral events. These findings support the feasibility of future real-time detection systems but do not constitute validation of a live predictive monitoring system. Prospective deployment and evaluation under operational classroom conditions will be necessary before conclusions can be drawn regarding real-world effectiveness. The integration of this framework into wearable technology aligns with Applied Behavior Analysis (ABA) by enhancing antecedent-based intervention strategies and complements Cognitive Behavioral Therapy (CBT) by providing objective indicators of internal emotional and physiological states that may not be externally observable. The findings extend theories of emotional regulation by suggesting that autonomic nervous system markers, particularly HRV and GSR, may function as nonverbal indicators of dysregulation and contribute to understanding internal antecedents associated with behavioral escalation (91). This perspective expands traditional behavioral approaches by incorporating physiological information that may precede observable behavior.

The study further demonstrates the feasibility of collecting wearable sensor data within naturalistic educational settings, providing a practical complement to laboratory-based assessment methods. While promising, additional research is needed to evaluate generalizability across diverse populations, settings, and educational contexts. Future investigations should focus on prospective validation, real-time implementation, ethical safeguards, and technical refinements necessary for sustainable deployment. Ultimately, this line of research offers a promising data-informed approach for supporting behavioral and emotional well-being among neurodiverse learners.

## Data Availability

The raw data supporting the conclusions of this article will be made available by the authors, without undue reservation.
